# Cardiac gating using scattering of an 8‐channel parallel transmit coil at 7T

**DOI:** 10.1002/mrm.27038

**Published:** 2017-12-11

**Authors:** Sven H.F. Jaeschke, Matthew D. Robson, Aaron T. Hess

**Affiliations:** ^1^ University of Oxford Centre for Clinical Magnetic Resonance Research, John Radcliffe Hospital Oxford United Kingdom

**Keywords:** parallel transmit, cardiac gating, motion sensor, cardiac MRI, reflected power, RF scattering

## Abstract

**Purpose:**

To establish a cardiac signal from scattering matrix or scattering coefficient measurements made on a 7T 8‐channel parallel transmit (pTx) system, and to evaluate its use for cardiac gating.

**Methods:**

Measurements of the scattering matrix and scattering coefficients were acquired using a monitoring pulse sequence and during a standard cine acquisition, respectively. Postprocessing used an independent component analysis and gating feature identification. The effect of the phase of the excitation radiofrequency (RF) field ( $B_{1}^{+}$ shim) on the cardiac signal was simulated for multiple $B_{1}^{+}$ shim configurations, and cine images were reconstructed from both the scattering coefficients and electrocardiogram (ECG).

**Results:**

The cardiac motion signal was successfully identified in all subjects with a mean signal‐to‐noise ratio of 33.1 and 5.7 using the scattering matrix and scattering coefficient measurements, respectively. The dominant gating feature in the cardiac signal was a peak aligned with end‐systole that occurred on average at 311 and 391 ms after the ECG trigger, with a mean standard deviation of 13.4 and 18.1 ms relative to ECG when using the scattering matrix and scattering coefficients measurements, respectively. The scattering coefficients showed a dependence on $B_{1}^{+}$ shim with some shim configurations not showing any cardiac signal. Cine images were successfully reconstructed using the scattering coefficients with minimal differences compared to those using ECG.

**Conclusion:**

We have shown that the scattering of a pTx RF coil can be used to estimate a cardiac signal, and that scattering matrix and coefficients can be used to cardiac gate MRI acquisitions with the scattering matrix providing a superior cardiac signal. Magn Reson Med 80:633–640, 2018. © 2017 The Authors Magnetic Resonance in Medicine published by Wiley Periodicals, Inc. on behalf of International Society for Magnetic Resonance in Medicine. This is an open access article under the terms of the Creative Commons Attribution License, which permits use, distribution and reproduction in any medium, provided the original work is properly cited.

## INTRODUCTION

Cardiovascular MRI at a magnetic field strength of 7T is a promising field of research 1, 2, 3, 4 but requires reliable, accurate, and user‐independent triggering and gating. The current gold‐standard gating method, based on the electrocardiogram (ECG), is affected by strong magnetic fields through magnetohydrodynamic effects 5. Whereas ECG gating is possible, lead re‐positioning is frequently required 2 and increases subject setup time. Missed or false trigger results can increase scan time or degrade image quality.

Different approaches have been developed to improve cardiac gating in high field (amplitude of static (polarizing) field (B_0_) ≥ 3T) MRI. Vector cardiographic gating enables more reliable trigger derived from the R‐wave of the ECG 6, 7. Self‐navigated sequences, coupled with multi‐channel receive arrays, have been used for automatic determination of the cardiac and respiratory motion 8, 9. Real‐time imaging acquisitions, which do not require cardiac gating, can be used 10. For more standard imaging acquisitions, external devices based on heart tones 11 or pilot tone signal modulation 12, 13, 14 were developed for cardiac gating. However, there remains a need for a cardiac gating method that does not require additional hardware or expert setup.

Buikman et al. 15 introduced a different approach to cardiac gating. They showed that the radiofrequency (RF) coil itself can be used to monitor motion as the quality factor of the coil, and therefore the fractional reflected power changes as a response to motion. Since then, a number of methods have been proposed to measure motion, primarily respiratory, by monitoring RF coils. These methods include monitoring of a single MR loop coil to detect cardiac and respiratory motion 16, and methods to estimate respiratory motion using pickup coils that monitor the complex currents in a parallel transmit (pTx) system 17, 18, thermal noise variance of a receiver array 19, and the scattering of a pTx coil to track the diaphragm position 20.

In this work, we extend the respiratory methods of reference 20, evaluating the sensitivity of the scattering matrix ( S) and the scattering coefficients ( $\vec{\Gamma }$) of an 8‐channel pTx coil for cardiac gating at 7T. The scattering matrix is measured using a dedicated pulse sequence. The scattering coefficients are both simulated from the scattering matrix measurements as well as measured directly from imaging RF pulses in a standard cine pulse sequence. We explore different potential gating features, compare the quality of the cardiac signal in the scattering matrix and scattering coefficients, and evaluate the influence of the phase of the excitation RF field ( $B_{1}^{+}$ shim) on the latter.

## THEORY

The theoretical basis of this technique was outlined in detail in reference 20. In brief, the pTx coil, which is electrically loaded by the human body, can be described as an N‐port electrical network in which N is the number of transmit channels of the pTx system. In the absence of measurement error, the NxN scattering matrix $S_{n,n}$ defines the fractional, returned voltage $V_{i,j,\text{ret}}\;$ on each channel *i*, which originates from the forward voltage ( $V_{j,\text{fwd}\;}$) on channel *j*:

(1) $$V_{i,j,\text{ret}}=S_{i,j}\;V_{j,\text{fwd}\;}.$$

This returned voltage arises due to mutual inductance between coil elements and the impedance mismatch between the characteristic impedance of the amplifier and the apparent impedance at each of the N input ports. These in turn are sensitive to the complex conductivity of the tissue within the field of view of each coil element. In the case of cardiac motion, the changing distribution of tissue and volume of highly conductive blood in the heart in different phases of the cardiac cycle leads to a change in the apparent impedance. We thus model the time‐dependent scattering matrix  S(t) with two terms (here neglecting respiratory motion): $\;S_{0}$, which is temporally invariant and represents the coil properties and static tissue, and $\Delta S_{\text{cardiac}}\left(t\right)$, which is the change resulting from cardiac motion:

(2) $$S\left(t\right)=S_{0}+\Delta S_{\text{cardiac}}\left(t\right).$$

## METHODS

### Setup

All experiments were carried out using a 7T MRI scanner (VB17, step 2.3, Siemens 7.0 T Magnetom, Erlangen, Germany) and an 8‐channel pTx system with a custom‐build transverse electromagnetic (TEM) cardiac transmit/receive array 21, with four elements placed anterior and four elements placed posterior. Eight healthy volunteers (male, age = 24–39 years) participated in this study. Directional couplers are built into each of the RF‐transmission lines as part of the pTx safety system to monitor the specific absorption rate. These directional couplers split off a small proportion of the complex RF signal and were used to quantify the forward ( ${\vec{V}}_{\text{fwd}}\left(t\right)$) and return ( ${\vec{V}}_{\text{ret}}\left(t\right)$) RF waveforms. These measurements are digitized using the standard MR receiver at a sampling rate of 1 MHz after demodulation of the system frequency. ECG trigger times were recorded from the MR scanner using a three‐lead commercial vector ECG module (Siemens).

### Scattering Matrix Measurements S

Frequency‐multiplexed pTx Gaussian profile RF‐pulses (frequency range 0–14 kHz in steps of 2 kHz per channel, amplitude = 11.1 V, pulse duration = 5 ms, repetition time (TR) = 10 ms) were used to obtain distinct waveforms of $\;{\vec{V}}_{\text{fwd}}\left(t\right)$ for each transmit channel 20. Multiple datasets (2–4 per participant) of the forward voltages $\;{\vec{V}}_{\text{fwd}}\left(t\right)$ and returned voltages ${\vec{V}}_{\text{ret}}\left(t\right)$ were recorded. Each subject was instructed to hold their breath for 15 seconds and then breath freely for a remaining 25 seconds. A fast Fourier transformation was used to identify each $V_{j,\text{fwd}}\left(t\right)$ and $\;V_{i,j,\text{ret}}\left(t\right)$ and thus to calculate  S(t) with Equation (1).

In one subject, the measurement was repeated after maintenance on the RF‐coil due to a subject‐specific failure.

### Scattering Coefficients $\vec{\Gamma }$

Typical MRI cine acquisitions at a field strength of 7T use a static $B_{1}^{+}$ shim, with all channels transmitting the same RF pulse simultaneously. S(t) cannot be determined during this kind of pulse sequence because the source of the returned voltages cannot be identified without changing the RF pulse or the sequence. However, ${\vec{V}}_{\text{ret}}\left(t\right)$ remains a function of $\Delta S_{\text{cardiac}}\left(t\right)$, and the directional couplers measure the forward voltages $\;{\vec{V}}_{\text{fwd}}\left(t\right)$, which are a function of the transmit voltage ( ${\vec{V}}_{\text{tx}}\left(t\right)$) and any noise or drift in the amplifiers ( $\vec{e}\left(t\right)$), as follows:

(3) $${\vec{V}}_{\text{ret}}\left(t\right)=\left(S_{0}+\Delta S_{\text{cardiac}}\left(t\right)\right)\left[\;{\vec{V}}_{\text{tx}}\left(t\right)+\vec{e}\left(t\right)\right],$$

where $\;{\vec{V}}_{\text{fwd}}\left(t\right)={\vec{V}}_{\text{tx}}\left(t\right)+\vec{e}\left(t\right)$. The static scattering ( $S_{0}$) can be determined before the scan by either a direct measurement of $\;S_{0}$, using the method in the previous section, or as in this work, by averaging the time series of  S(t). Then, $S_{0}$ can be used to normalize ${\vec{V}}_{\text{ret}}\left(t\right)$ and correct amplifier drifts with an element wise vector division to form a vector of scattering coefficients $\vec{\Gamma }\left(t\right)$ that are sensitive to motion 20:

(4) $$\vec{\Gamma }\left(t\right)=\frac{{\vec{V}}_{\text{ret}}\left(t\right)}{S_{0}{\vec{V}}_{\text{fwd}}\left(t\right)}=1+\frac{\Delta S_{\text{cardiac}}\left(t\right){\vec{V}}_{\text{fwd}}\left(t\right)}{S_{0}{\vec{V}}_{\text{fwd}}\left(t\right)}.$$

### $B_{1}^{+}$ Shim and Scattering Coefficients ${\vec{\Gamma }}^{A}$

To facilitate a direct comparison between the cardiac signal derived from S(t) and that from $\;\vec{\Gamma }\left(t\right)$, scattering coefficients ${\vec{\Gamma }}^{A}\left(t\right)$ were simulated from the above measurements of  S(t) during breath hold, which additionally enabled evaluation of the effects of $B_{1}^{+}$ shimming. To evaluate the effects of $B_{1}^{+}$ shimming, 1,000 vectors ( $\vec{A}$) with components of unit magnitude and random uniformly distributed phases were created for each dataset  S(t). These vectors ( $\vec{A}$) were retrospectively applied to each of the frequency de‐multiplex ${\vec{V}}_{fwd}\left(t\right)$ (that have been used to determine  S(t)) with an element‐wise multiplication to give $B_{1}^{+}$ shim‐dependent forwarded voltages $\;{\vec{V}}_{fwd}^{A}\left(t\right)$. The corresponding returned voltages were calculated as $\;\;{\vec{V}}_{ret}^{A}\left(t\right)=S\left(t\right)\;{\vec{V}}_{fwd}^{A}\left(t\right)$. For each $B_{1}^{+}$ shim and S(t) dataset, the scattering coefficients ${\vec{\Gamma }}^{A}\left(t\right)$ were determined using Equation (4), where $S_{0}\;$ was taken as the average of  S(t).

### Scattering Coefficients $\vec{\Gamma }$ Measurement in Retrospective Cine

A 2D, retrospectively gated, cine gradient recalled echo sequence was acquired in five of the participants. The parameters were voxel size = 1.3 × 1.3 × 6 mm, TR = 29 ms, echo time = 2 ms, flip angle ≈ 10°, generalized autocalibrating partially parallel acquisitions factor of two with 24 reference lines, and total scan time of approximately 18 s. $\vec{\Gamma }\left(t\right)$ was calculated for each RF pulse during the acquisition without the use of any additional monitoring pulses or pulse sequence modifications. $S_{0}\;$ was acquired prior to the imaging scan by averaging the time series S(t), which was measured as described above. The cardiac signal was calculated from $\vec{\Gamma }\left(t\right),\;$ and peak detection was carried out as described below. The k‐space data was binned by either using the scattering or ECG. The images were reconstructed with the PULSAR MatLab toolbox V1.1 (MathWorks, Natick, MA, USA) 22.

### Cardiac Signal Extraction

All signal analysis was conducted retrospectively in MatLab (MathWorks). To extract the cardiac signal, the data was separated into real and imaginary components, giving 128 (8 × 8 × 2,  S(t)) or 16 (8 x 2, $\;\vec{\Gamma }\left(t\right)$) data points for each measurement forming a measurement vector $\;\vec{x}\left(t\right)$, which was analyzed automatically according to the following five steps:

1. Demeaning and de‐trending to remove any drifts using quadratic polynomials fitted and subtracted from the data
2. Low‐pass filtering using a digital Butterworth filter object 23 to eliminate spikes and noise (infinite impulse response design with an order of 17, passband frequency 7.5 Hz, stopband frequency 15 Hz). The filter was applied in a forward and backward fashion to avoid time shifts in the filtered signal.
3. Principal component analysis and independent component analysis using Hyvärinen's fixed‐point algorithm 24 built into the FastICA V2.5 25) software package for MatLab (MathWorks). The dimensionality reduction was based on the eigenvalues of the covariance matrix; values greater than an absolute threshold of *ε* = 0.75 × 10^−7^(default *ε* = 1 × 10^−7^) were kept; and the dimension was reduced to the number of kept eigenvalues.
4. A Welch power spectrum density estimation was used to identify the correct cardiac component automatically by finding the maximum power within the cardiac frequency range of 0.6 Hz to 2.4 Hz of all estimated components. This information was used to determine the cardiac de‐mixing vector and to extract the cardiac signal c(t) from the measurements $\vec{x}\left(t\right)$.
5. The polarity of the cardiac signal was standardized to enable automatic signal analysis. This was determined using a peak detection on the absolute signal, followed by a k‐means clustering with the corresponding peak height.

### Gating Features From Cardiac Signal

The cardiac signal  c(t)  was used to establish four further traces to facilitate peak detection and to assess different gating features. Two traces were derived from a multilevel, discrete wavelet transform with a maximal overlap 26 and a symlet wavelet of order 5. For the first trace, which is denoted as $c_{w6}\left(t\right)$, only the wavelet coefficients that correspond to the expected frequency range of the heart rate were kept. In addition, an inverse wavelet transform was performed (level = 6), which ensured a single peak per cardiac cycle. The second trace, $c_{w4}\left(t\right)$, was established with an inverse wavelet transform with a higher frequency window (level = 4), which resulted in two peaks per cardiac cycle.

The third and fourth traces were created by applying a Savitzky‐Golay filter 27 on the cardiac signal c(t), followed by a calculation of the first‐ and second‐order discrete temporal derivatives and denoted as c′(t) and c″(t), respectively. For all traces, the local maxima were identified using MatLab (MathWorks), and the resulting peaks were separated, when necessary, by using k‐means clustering on the cardiac signal and the corresponding traces. These traces led to five different gating features, which were assessed in a retrospective signal analysis when using  S(t). In the cardiac signal, based on measurements of $\;\vec{\Gamma }\left(t\right)$, only the main peak of the first trace was apparent, and it was the only gating feature evaluated.

The noise in the cardiac signal of each dataset was calculated by subtracting the filtered (Savitzky‐Golay filter 27) cardiac signal from the unfiltered cardiac signal and taking this standard deviation (SD) as the magnitude of noise. The mean peak amplitude of the cardiac signal, divided by this noise, is the signal‐to‐noise ratio (SNR) of the cardiac signal.

### Evaluation of Gating Accuracy

Statistical evaluation was carried out for breath hold and free breathing separately. Cardiac cycles with ectopic beats or missed ECG triggers were manually excluded. The delays between the ECG trigger ( $R_{n}$) and the detection time of the respective feature ( $F_{n}$), detected in cardiac cycle *n*, were averaged to determine a mean trigger delay (*MTD*):

(5) $$MTD=\frac{1}{N}\overset{N}{\underset{n=1}{\sum }}\left(F_{n}-R_{n}\right)\;,$$

where N is the total number of cardiac cycles. Here, a negative *MTD* corresponds to a peak detection before the ECG trigger event. The SD s of the time difference was also calculated:

(6) $$s=\;\sqrt{\frac{1}{N-1}\overset{N}{\underset{n=1}{\sum }}{\left(\;\left(F_{n}-R_{n}\right)-MTD\right)}^{2}\;}.$$

The peak detection performance was evaluated using the sensitivity (*Se*) and the positive predictive value (*PPV*), which were calculated with Equations (7) and (8), respectively. Each of the gating features were assumed to occur exactly one time during a normal heart cycle, and were manually assessed by overlaying the ECG trigger events and the extracted cardiac signal and counting the number of detected features. Whereas true positives (*TP*) were correctly identified events, false negatives (*FN*) were missed events and additional peaks (including in the ECG) were counted as a false positive (*FP*).

(7) $$Se=\;\frac{TP}{TP+FN}$$

(8) $$PPV=\frac{TP}{TP+FP}.$$

## RESULTS

The identification of the cardiac component failed on one dataset, which has been excluded and replaced with data from a second scan on the same subject when it succeeded. This failure was due to a coil failure. The cardiac signal was identified automatically in all remaining datasets when using  S(t) and $\;\vec{\Gamma }\left(t\right)$.

**Figure 1:**
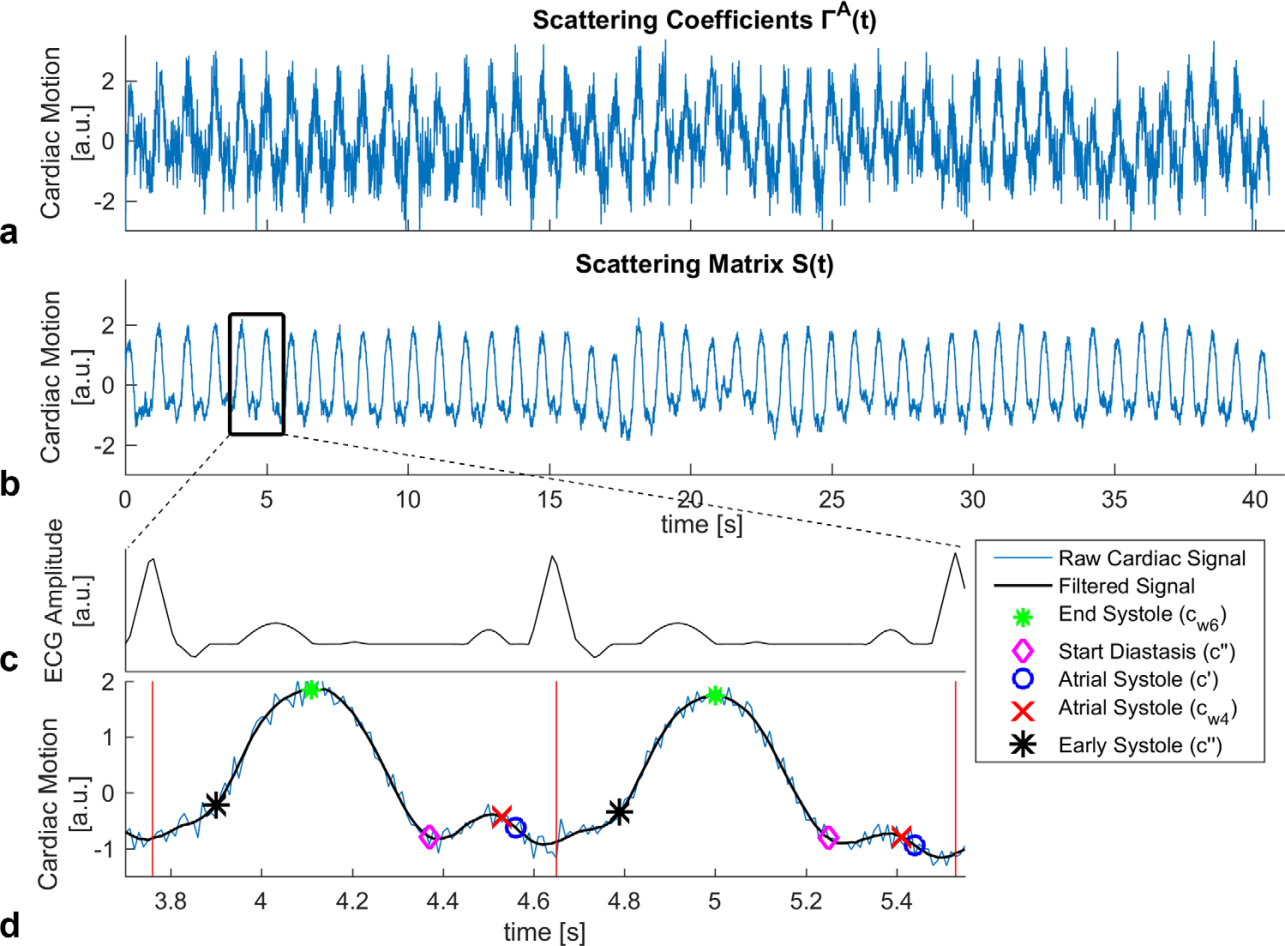
The extracted cardiac signal is shown using (**a**) the scattering coefficients $\vec{\Gamma }\left(t\right)$ and (**b**) the scattering matrix  S(t). (**c–d**) Two selected cardiac cycles are shown and compared with an artificial ECG trace. The different features are displayed on the cardiac signal extracted using  S(t). The end‐systole is targeted with the main peak (green star) of c_w6._ The feature that coincides with the start of the diastasis is indicated by a magenta diamond (c″), those for atrial contraction by blue circles and red crosses (targeted by using c′ and c_w4_, respectively), and the feature for the early systole (c″) by black stars. Vertical red lines are ECG trigger time points (R‐peak). ECG, electrocardiogram.

Figure 1 shows an example cardiac signal extracted using S(t) and ${\vec{\Gamma }}^{A}\left(t\right),\;$ as simulated with an appropriate $B_{1}^{+}$ shim from the same dataset, with one large peak per heart cycle that occurs close to the time of end‐systole. The SNR of the cardiac signal had a mean value of 33.1 (11.4–45.7) using S(t)  and a mean value of 5.7 (2.4–10.2) using $\;\vec{\Gamma }\left(t\right)$, as measured during cine acquisition.

**Figure 2:**
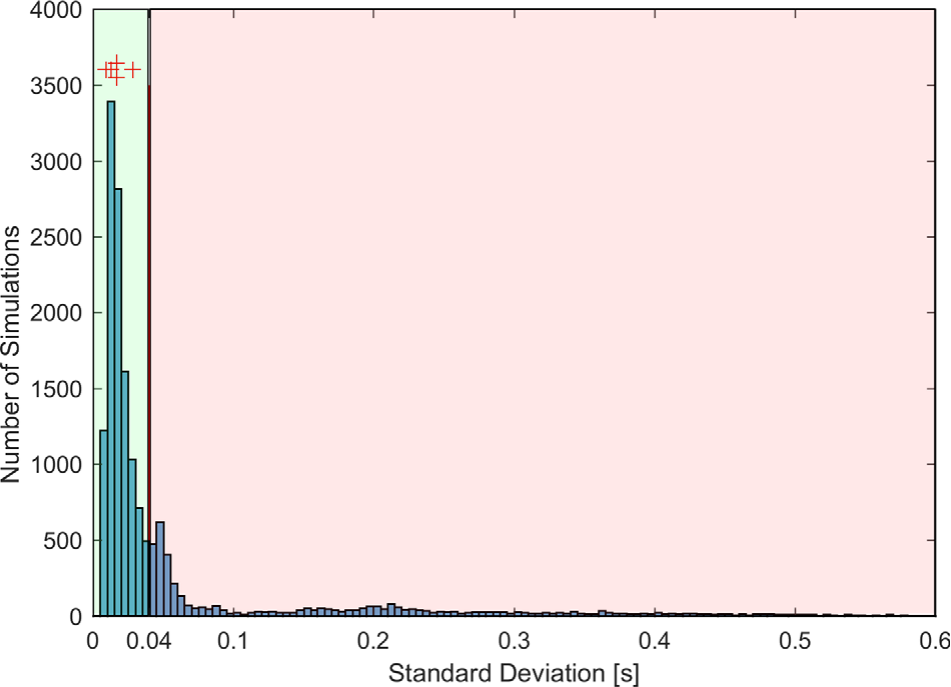
Histogram of the SD of the difference in trigger time between the ECG (R‐wave) and the main peak detection (end‐systole), derived from the cardiac signal using the synthesized scattering coefficients ${\vec{\Gamma }}^{A}\left(t\right)$, is shown. The quality of the end‐systole detection varies with the $B_{1}^{+}$ shim, with the majority of shim sets having a low SD compared to ECG ( < 40 ms). The results using all synthesized datasets (n = 16000) during breath hold are shown in the histogram. Red crosses indicate the results from the cardiac signal derived from Cine images using the scattering coefficients $\vec{\Gamma }\left(t\right)$ (n = 5). $B_{1}^{+}$ = amplitude of (excitation) radiofrequency field, ECG, electrocardiogram; SD, standard deviation.

Figure 2 shows the effect of changing $B_{1}^{+}$ shim on the accuracy of the detection of end‐systole in the simulated scattering coefficients ${\vec{\Gamma }}^{A}\left(t\right)$, using a histogram of the SD (relative to ECG). Assuming that a SD of greater than 40 ms limits the suitability for cardiac MRI, the figure shows that 71% of random $B_{1}^{+}$ shim combinations were successful and also showed sufficient cardiac information available in $\;{\vec{\Gamma }}^{A}\left(t\right)$. For these successful combinations, the end‐systolic peak was detected with a sensitivity of 98.9%, a positive predictive value of 98.5%, a mean trigger delay of 391 ms after the ECG R‐wave, and a mean SD of 18.1 ms relative to ECG. Using the cardiac signal from the scattering matrix S(t), the detection of the end‐systole was the most reliable (Se and PPV = 100%) and least variable during breath hold, with a mean trigger delay of 311 ms after the ECG R‐wave and a mean SD of 13.4 ms relative to ECG.

For further comparison of the cardiac signal derived from S(t) and that from $\vec{\Gamma }\left(t\right)$, measured during cine acquisitions, a Bland‐Altman analysis (31) was carried out to compare the peak‐to‐peak interval of the end‐systole feature with the peak‐to‐peak (R‐R) interval of the ECG. These analyses are plotted in https://onlinelibrary.wiley.com/action/downloadSupplement?doi=10.1002%2Fmrm.27038&attachmentId=211523122 (available online). They demonstrated that the peak‐to‐peak interval was within −27/+24 ms and within −50/+52 ms of that calculated with ECG on a 96% confidence interval for S(t)  and $\;\vec{\Gamma }\left(t\right),\;$ respectively.

Aside from the main peak of the first trace, which occurs near the time of end‐systole, four further gating features were identified that occur near the following phases of the heart cycle:

1. Atrial contraction, identified as a second, minor peak in $c_{w4}\left(t\right)$
2. Atrial contraction, identified as the small, negative peak in $c^{\prime \left(t\right)}$
3. Early systole, close to the ECG R‐wave and start of ventricular contraction, identified as the peak in c″(t) and
4. Start of the diastasis, when the heart is almost at rest and where least heart motion occurs, identified as peak in $c^{\prime \prime \left(t\right)}$ and minima of $\;c^{\prime \left(t\right)}$

**Figure 3:**
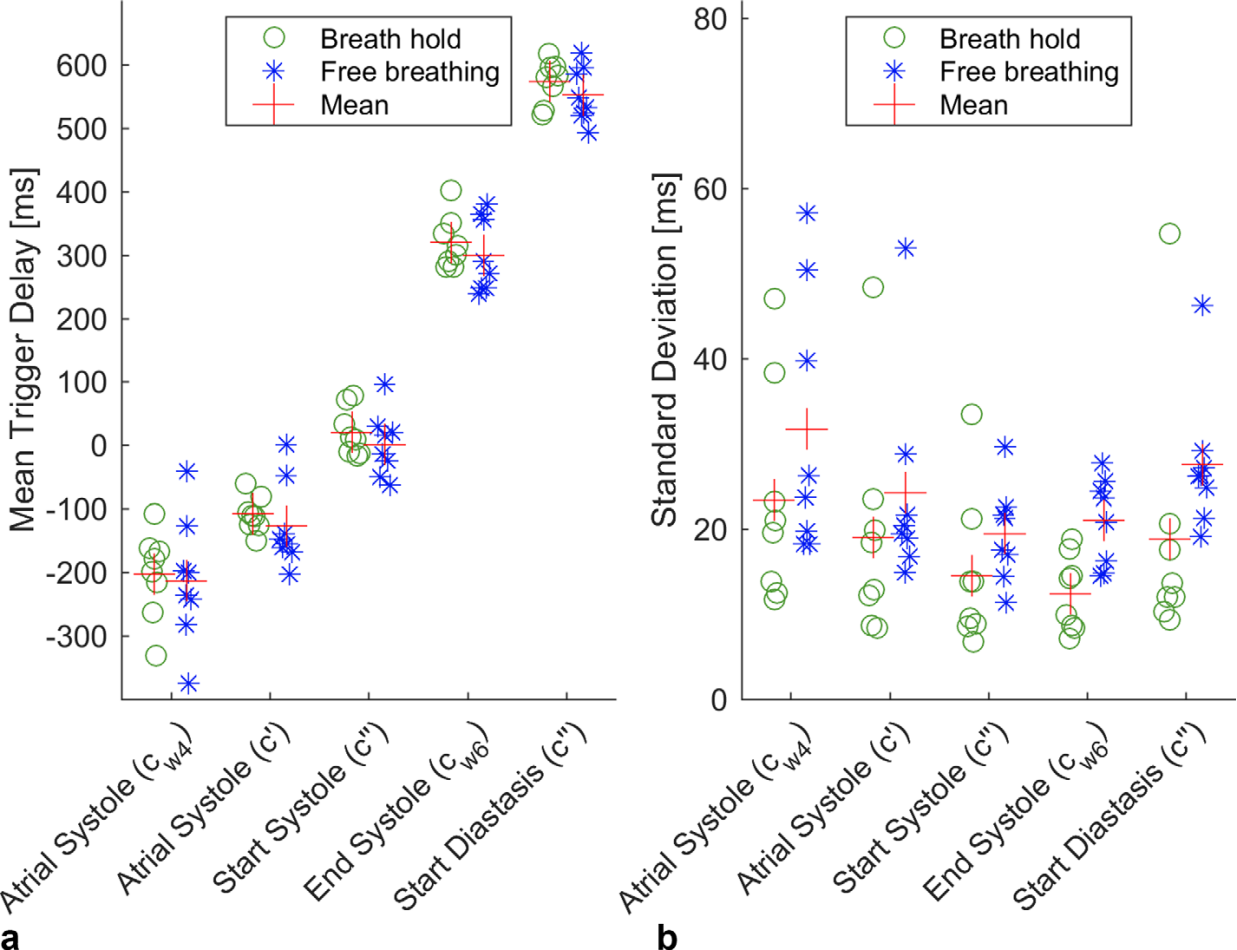
(**a**) Intrasubject mean trigger delay and (**b**) SD of the difference between the gating features and electrocardiogram trigger identified on the cardiac signal using the scattering matrix  S(t). The data is grouped by different features and breath hold/free breathing. The lowest mean SD is shown by the main peak detection (c_w6_) with 13.4 ms during breath hold, whereas the gating feature, which occurs closest to the atrial systole, shows the highest mean SD. SD, standard deviation.

Figure 3 plots the mean trigger delay and SD of the difference to ECG (R‐peak) for each of the five gating features detected on the cardiac signal using  S(t). The variability relative to ECG increased in all features during free breathing. The sensitivity and positive predictive values for all gating features are listed in Table 1.

**Table 1:** Summary Statistics for Gating Features

Feature ID	Breath Hold/Free Breathing	Sensitivity [%]	Positive Predictive Value [%]	Mean Trigger Delay [ms]	Mean Standard Deviation of the Difference to ECG [ms]
Atrial Systole $c_{w4}\left(t\right)$	Breath hold	97.7	98.0	−215	26.3
	Free breathing	93.5	97.6	−227	33.8
Atrial Systole $c^{\prime \left(t\right)}$	Breath hold	96.0	96.8	−114	22.5
	Free breathing	92.4	95.7	−137	27.5
Start Systole c″(t)	Breath hold	100.0	100.0	22	16.7
	Free breathing	99.6	100.0	−0.5	21.1
End Systole $c_{w6}\left(t\right)$	Breath hold	100.0	100.0	311	13.4
	Free breathing	100.0	100.0	288	21.9
Start Diastasis $c^{\prime \prime \left(t\right)}$	Breath hold	97.4	100.0	570	22.9
	Free breathing	96.1	98.3	541	30.2
End Systole ( $c_{w6}\left(t\right))$ (scattering coeff. $\vec{\Gamma }\left(t\right)$)	Breath hold	99.8	100.0	391	18.1

ECG, electrocardiogram.

**Figure 4:**
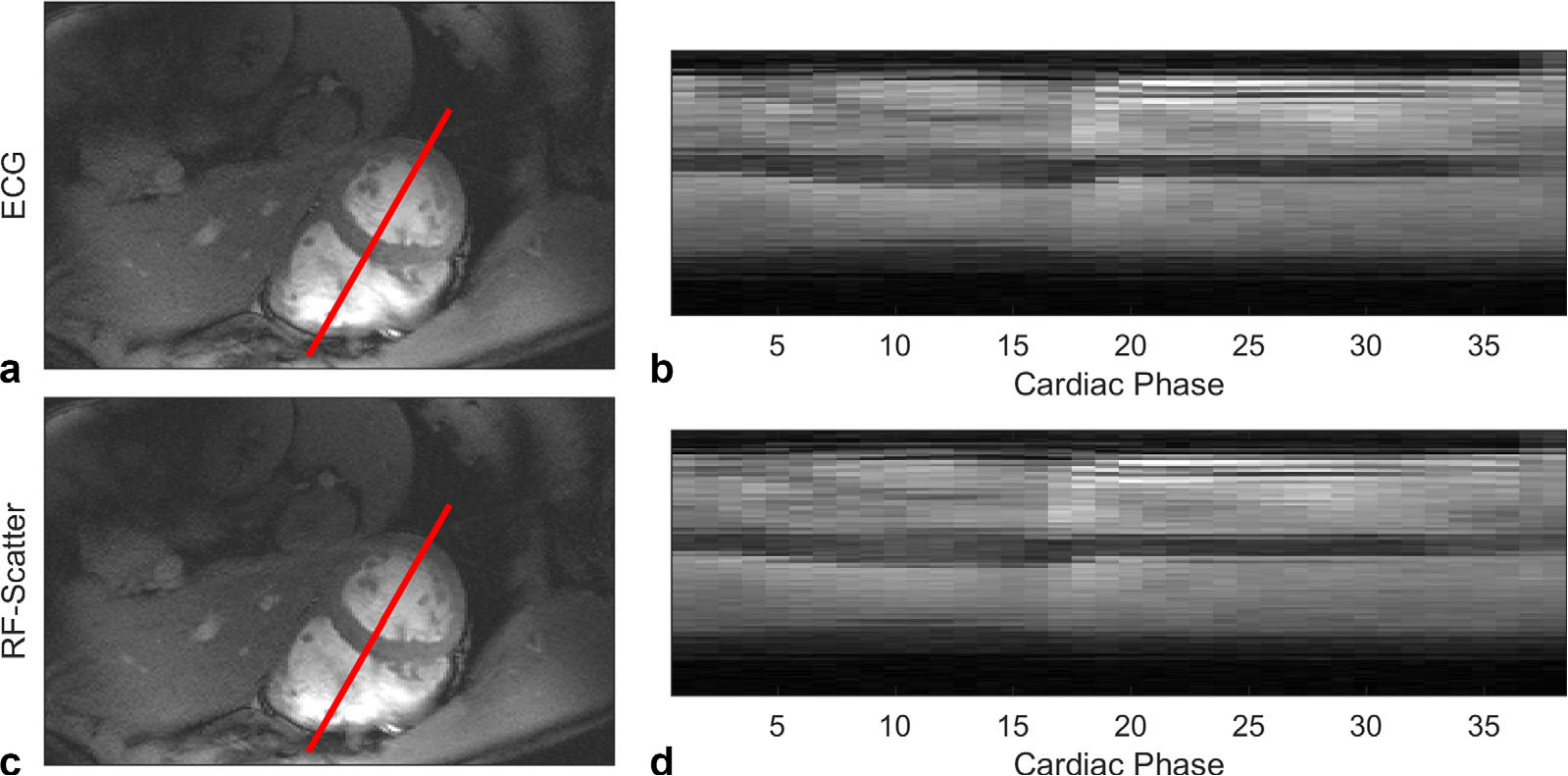
Short‐axis, midventricular slice (**a**, **c**), and temporal profiles (**b**, **d**) of a breath hold CINE gradient‐recalled echo sequence reconstructed using the ECG trigger (**a**, **b**) and scattering coefficients (**c**, **d**) measured during transmit RF‐pulses with main peak detection (end‐systole), with ∼10° flip angle, 8 k‐space lines per phase, repetition time = 29 ms acquired with a fixed 37 phases, and total scan time of 18 s. For comparison reasons, the trigger delay of the RF‐scatter triggering to ECG was corrected to align both Cine images. ECG, electrocardiogram; RF, radiofrequency.

Retrospective cardiac gating, using $\;\vec{\Gamma }\left(t\right)$, was tested successfully in cines on five subjects with minimal differences compared to those reconstructed from ECG. The SD of the difference in main peak detection time compared to ECG are plotted as the markers in the histogram of Figure 2. Figure 4 compares the images that were reconstructed using ECG and $\;\vec{\Gamma }\left(t\right)$ for one subject, whereas the four remaining cine datasets are shown in https://onlinelibrary.wiley.com/action/downloadSupplement?doi=10.1002%2Fmrm.27038&attachmentId=211523122 (available online). https://onlinelibrary.wiley.com/action/downloadSupplement?doi=10.1002%2Fmrm.27038&attachmentId=211523123 to https://onlinelibrary.wiley.com/action/downloadSupplement?doi=10.1002%2Fmrm.27038&attachmentId=211523127 (available online) also provide movies of the reconstructed cines from all subjects compared to ECG gating.

## DISCUSSION

We have shown that a cardiac signal can be extracted from the scattering of a pTx RF coil at 7T MRI by measuring either the scattering matrix S(t) or the scattering coefficients $\;\vec{\Gamma }\left(t\right)$.

The cardiac signal extracted using  S(t) shows a greater SNR compared to the cardiac signal extracted using $\;\vec{\Gamma }\left(t\right)$. For  S(t), the end‐systolic peak was detected with 100% sensitivity and positive predictive value. The higher SNR, when using  S(t), enables identification of multiple features in the cardiac cycle. These other gating features aligned with early systole, start of diastasis, and atrial contraction. The start of the diastasis shows a good overall performance and can provide additional information about the heart state compared to ECG. In subjects with high mean heart rates ( > 80 beats per minute), the diastasis feature has a lower sensitivity (mean Se = 88%), which may result from a reduced duration of the resting time after passive refilling. The detection of the atrial contraction (corresponding P‐wave in the ECG), has the lowest sensitivity and highest SD compared to ECG gating, whereas the use of wavelets leads to a more robust but less accurate identification than using first‐order derivatives. The feature that aligns with the early systole has a high sensitivity and a good positive predictive value and was only outperformed by the main peak at end‐systole. However, compared to end‐systole, it aims to detect a rather small change in the cardiac signal and thus is more difficult to detect.

All gating features show a higher variance during free breathing than in breath hold (see Fig. 3). Comparing the cardiac signal during inspiration and expiration, small changes can be observed that affect the feature detection. These subtle changes may arise from genuine physiological differences in cardiac function during respiration, such as a prolonged right‐sided ejection time and increased venous return during inspiration 29.

The gating accuracy, when using the simulated scattering coefficients $\;{\vec{\Gamma }}^{A}\left(t\right),$ was dependent on the choice of $B_{1}^{+}$ shim, with a large subset of $B_{1}^{+}$ shims providing a gating signal suitable for cine imaging. This dependency may be due to a phase cancellation effect of the resulting scattering. Further work is required to understand the relationship between $B_{1}^{+}$, lead field superposition 30, and the cardiac signal.

Nonetheless, retrospectively gated cines were successfully reconstructed using $\;\vec{\Gamma }\left(t\right)$, which were measured during image acquisition, with no care in choice of $B_{1}^{+}$ shim other than a good $B_{1}^{+}$ field distribution over the heart 31. These reconstructed cines demonstrate that using $\vec{\Gamma }\left(t\right)$ is sufficient for gating purposes and is elegant to use because no sequence or hardware modification is required. However, the cardiac signal from the scattering matrix remains superior because it outperformed that from the scattering coefficient, and the peak‐to‐peak interval was more closely aligned to that from the ECG. These improvements in gating using S(t) compared to $\vec{\Gamma }\left(t\right)\;$ are likely a result of the greater SNR of the cardiac signal derived from  S(t). For S(t) to be useful in practice, fast methods of measuring will be required that minimally decrease the pulse sequence efficiency.

The measurement on volunteer 2 was acquired twice due to an unexpected systematic error in the first dataset. This error resulted in an overlaying frequency at exactly 1 Hz in the data, which disabled the extraction of the cardiac signal using the independent component analysis, and which disappeared after maintenance of the coil. This demonstrates the sensitivity of this method to the hardware state, and that more sensitive quality‐control methods need to be developed to detect faulty hardware. The cardiac signal may be sensitive to the type of RF coil, number of elements, and number of pTx channels that are used. More work is required to quantify those dependencies and evaluate the applicability in lower field (1.5T and 3T MRI).

## CONCLUSION

We have presented a retrospective cardiac gating method at 7T MRI, using scattering of a pTx coil and an independent component analysis. The scattering coefficient was outperformed by the scattering matrix, which was able to detect multiple gating features. This new approach has the potential to remove the requirement for cumbersome ECG setup and preparation time in cine imaging at 7T.

## Supporting information


**Fig. S1**. Intervals of the main peak detection (end‐systole), derived from the cardiac signal using the scattering matrix ***S***(*t*) from all datasets during breath hold (a‐b), and from the cardiac signal using the scattering coefficients $\vec{\Gamma }\left(t\right)$ measured during cine acquisitions (c‐d), compared to ECG intervals. (a,c) Scatter plot of peak‐to‐peak (RR)‐ECG interval and the peak‐to‐peak feature interval. (b,d) The Bland‐Altman plot reveals that the end systole detection occurs within a range of +24/‐27 ms using the scattering matrix (b) and within a range of +52/−55 ms using the scattering coefficients $\vec{\Gamma }\left(t\right)$ (d) compared to ECG intervals.
**Fig. S2**. Short‐axis, mid‐ventricle slice (diastole) and temporal profiles of all breath hold cine sequences reconstructed using the scattering coefficients, which were measured during transmit RF‐pulses, are shown. The main peak detection (end‐systole) of the proposed method was used for cardiac gating and retrospective re‐binning of the k‐space data.

**Video S1**. Short‐axis, mid‐ventricle slice of a breath hold cine sequence on volunteer 1. Cine images were reconstructed using either the ECG (left) or the scattering coefficients (right) that were measured during transmit RF‐pulses. The main peak detection (end‐systole) of the proposed method was used for cardiac gating and retrospective re‐binning of the k‐space data.

**Video S2**. Short‐axis, mid‐ventricle slice of a breath hold cine sequence on volunteer 2. Cine images were reconstructed using either the ECG (left) or the scattering coefficients (right) that were measured during transmit RF‐pulses. The main peak detection (end‐systole) of the proposed method was used for cardiac gating and retrospective re‐binning of the

**Video S3**. Short‐axis, mid‐ventricle slice of a breath hold cine sequence on volunteer 3. Cine images were reconstructed using either the ECG (left) or the scattering coefficients (right) that were measured during transmit RF‐pulses. The main peak detection (end‐systole) of the proposed method was used for cardiac gating and retrospective re‐binning of the

**Video S4**. Short‐axis, mid‐ventricle slice of a breath hold cine sequence on volunteer 4. Cine images were reconstructed using either the ECG (left) or the scattering coefficients (right) that were measured during transmit RF‐pulses. The main peak detection (end‐systole) of the proposed method was used for cardiac gating and retrospective re‐binning of the k‐space data.

**Video S5**. Short‐axis, mid‐ventricle slice of a breath hold cine sequence on volunteer 5. Cine images were reconstructed using either the ECG (left) or the scattering coefficients (right) that were measured during transmit RF‐pulses. The main peak detection (end‐systole) of the proposed method was used for cardiac gating and retrospective re‐binning of the k‐space data.
