# Supplementary material for: Cardiac gating using scattering of an 8‐channel parallel transmit coil at 7T
Source: Magn Reson Med. 2017 Dec 11;80(2):633–40. doi: 10.1002/mrm.27038 (PMC5947608; doi:10.1002/mrm.27038)

FIG. S1: Intervals of the main peak detection (end-systole), derived from the cardiac signal using the scattering matrix$\boldsymbol{S}\left( t \right)$ from all datasets during breath hold (**a-b**), and from the cardiac signal using the scattering coefficients $\vec{\Gamma}\left( t \right)$ measured during cine acquisitions (**c-d**), compared to ECG intervals. (**a,c**) Scatter plot of peak-to-peak (RR)-ECG interval and the peak-to-peak feature interval. (**b,d**) The Bland-Altman plot reveals that the end systole detection occurs within a range of +24/-27 ms using the scattering matrix (**b**) and within a range of +52/-55 ms using the scattering coefficients $\vec{\Gamma}\left( t \right)$ (**d**) compared to ECG intervals.


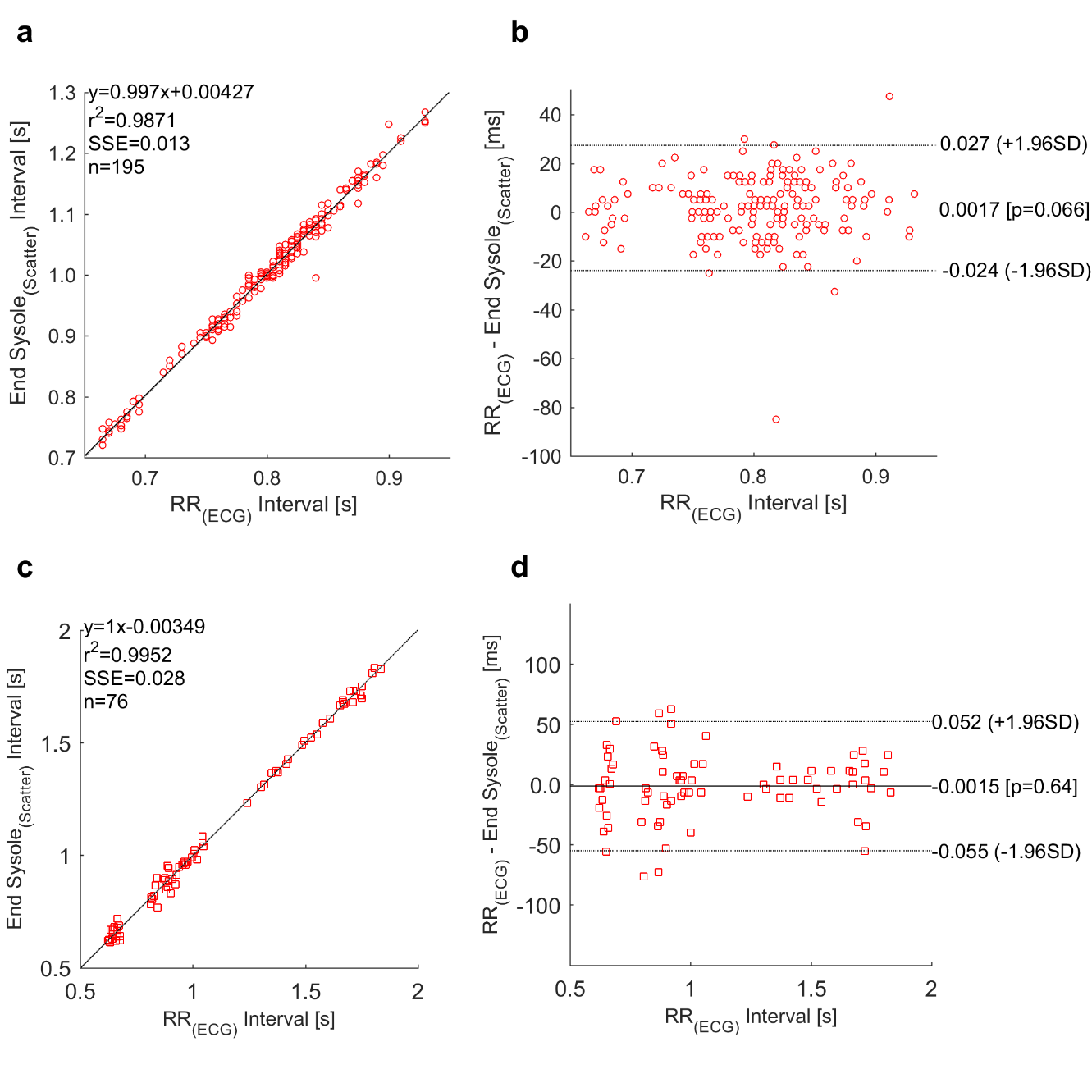


FIG. S2: Short-axis, mid-ventricle slice (diastole) and temporal profiles of all breath hold cine sequences reconstructed using the scattering coefficients, which were measured during transmit RF-pulses, are shown. The main peak detection (end-systole) of the proposed method was used for cardiac gating and retrospective re-binning of the k-space data.


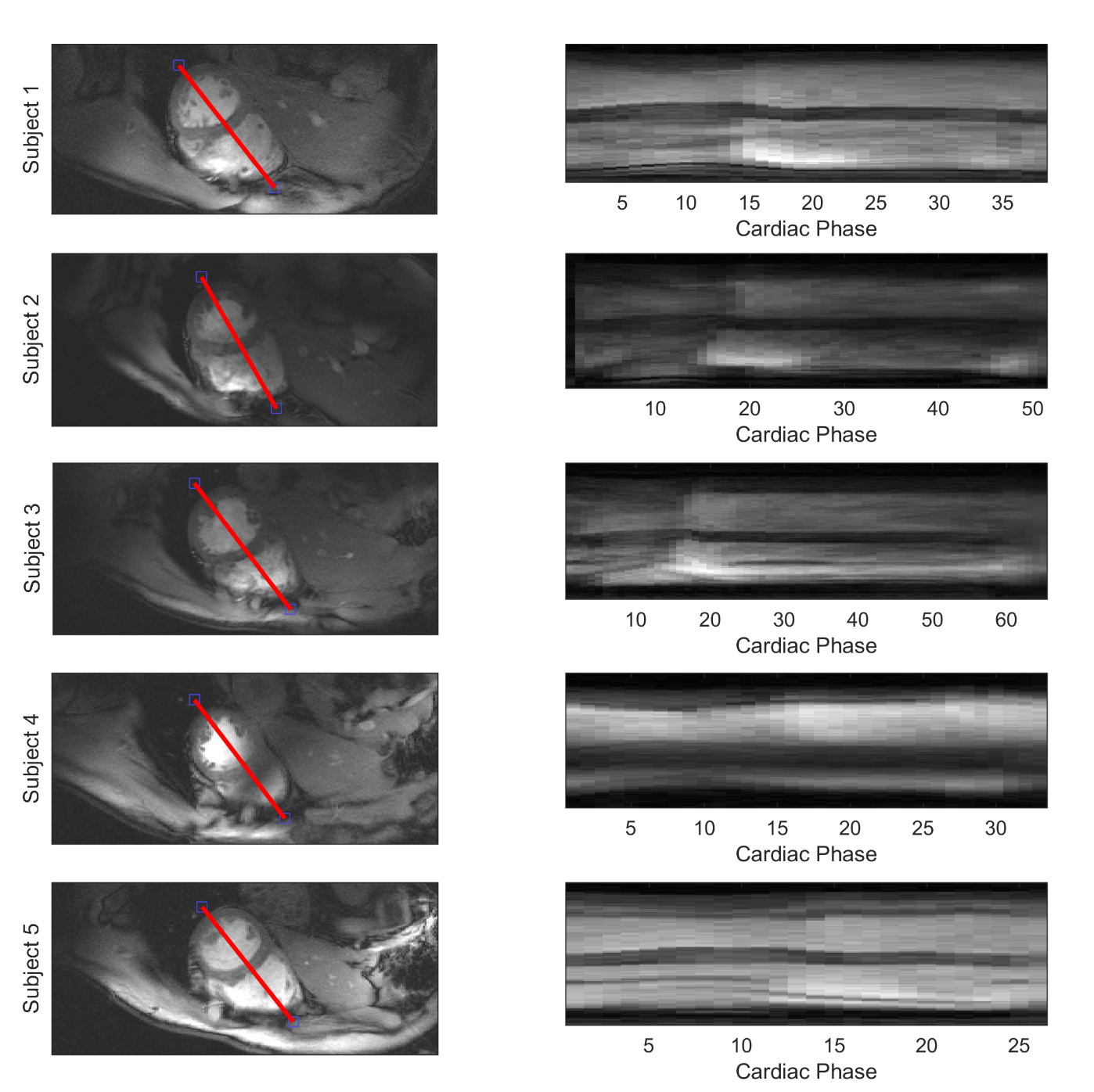

Supplement: Supplementary file 1 — Fig. S1. Intervals of the main peak detection (end‐systole), derived from the cardiac signal using the scattering matrix S(t) from all datasets during breath hold (a‐b), and from the cardiac signal using the scattering coefficients Γ→(t) measured during cine acquisitions (c‐d), compared to ECG intervals. (a,c) Scatter plot of peak‐to‐peak (RR)‐ECG interval and the peak‐to‐peak feature interval. (b,d) The Bland‐Altman plot reveals that the end systole detection occurs within a range of +24/‐27 ms using the scattering matrix (b) and within a range of +52/−55 ms using the scattering coefficients Γ→(t) (d) compared to ECG intervals. Fig. S2. Short‐axis, mid‐ventricle slice (diastole) and temporal profiles of all breath hold cine sequences reconstructed using the scattering coefficients, which were measured during transmit RF‐pulses, are shown. The main peak detection (end‐systole) of the proposed method was used for cardiac gating and retrospective re‐binning of the k‐space data. [file MRM-80-633-s001.docx]
